# MBOSS: A Symbolic Representation of Human Activity Recognition Using Mobile Sensors

**DOI:** 10.3390/s18124354

**Published:** 2018-12-10

**Authors:** Kevin G. Montero Quispe, Wesllen Sousa Lima, Daniel Macêdo Batista, Eduardo Souto

**Affiliations:** 1 Computer Institute, Federal University of Amazonas, Manaus 69080-900, Brazil; wesllen@icomp.ufam.edu.br (W.S.L.); esouto@icomp.ufam.edu.br (E.S.); 2 Department of Computer Science, University of São Paulo, São Paulo 05508-090, Brazil; batista@ime.usp.br

**Keywords:** human activity recognition, symbolic representation, inertial sensors, smartphone

## Abstract

Human activity recognition (HAR) through sensors embedded in smartphones has allowed for the development of systems that are capable of detecting and monitoring human behavior. However, such systems have been affected by the high consumption of computational resources (e.g., memory and processing) needed to effectively recognize activities. In addition, existing HAR systems are mostly based on supervised classification techniques, in which the feature extraction process is done manually, and depends on the knowledge of a specialist. To overcome these limitations, this paper proposes a new method for recognizing human activities based on symbolic representation algorithms. The method, called “Multivariate Bag-Of-SFA-Symbols” (MBOSS), aims to increase the efficiency of HAR systems and maintain accuracy levels similar to those of conventional systems based on time and frequency domain features. The experiments conducted on three public datasets showed that MBOSS performed the best in terms of accuracy, processing time, and memory consumption.

## 1. Introduction

In recent years, human activity recognition (HAR) has been used to understand how humans act, make decisions, and interact with other individuals [1]. For this reason, the HAR area has advanced towards the development of systems that are capable of identifying human behavior patterns from sensor data. These patterns, related to activities or habits, allow for the development of innovative applications that aid users and support decisions in different areas, such as health [2,3], security [4], and industry [5,6]. From this perspective, solutions for HAR based on data extracted from inertial sensors (e.g., accelerometer and gyroscope) embedded in smartphones have been widely explored in the literature [7,8,9]. In general, these sensors are used to recognize physical activities such as walking, running, sitting, standing, lying down, and walking up- and down- stairs [10]. Inertial sensors data is typically processed through the following steps: data collection, data segmentation, feature extraction, feature selection, and classification model training [11]. Among these steps, feature extraction is the most studied, since it directly impacts the performance of classification models [7].

In HAR based on inertial sensors signals, features are divided into two domains: the time domain (e.g., mean, variance and standard deviation), and the frequency domain (e.g., energy and spectral entropy) [8]. Although this approach can generate good classification models, this solution depends on human knowledge to determine a set of features that achieves significant improvements in computational performance and energy efficiency. In addition, this approach generates classification models which are dependent on the specific domains limited by the training databases [12]. To overcome this limitation, HAR systems have used deep learning algorithms to automatically extract the features. Although this strategy has achieved high accuracy rates and good generalization for the classification models, the computational cost required is unfeasible for smartphones with limited processing, memory, and battery resources. Efficient deep learning models for mobile devices as MobileNets [13] are intended to make this feasible in the future.

In an attempt to solve this problem, this article proposes a low-cost solution capable of automatically extracting features using a symbolic representation algorithm called “Symbolic Fourier Approximation” (SFA) [14]. These algorithms estimate the real values of a time series by transforming them into a chain of symbols or words. Each word represents a pattern or feature found in the data, and a set of words can be associated with a user’s activity. In addition, the classification models are based on the word frequencies distribution using the Bag-Of-Words or histograms that are commonly used in Information Retrieval (IR) [15].

The proposed solution is a symbolic classification algorithm called “MBOSS” (Multivariate Bag-Of-SFA-Symbols). MBOSS is adapted to manipulate three-dimensional data extracted from inertial sensors. MBOSS is an extension of the BOSS VS (SFA-Symbols in Vector Space) [16], with the main difference being that MBOSS is able to process multiple time series using a histogram fusion strategy. In addition, in the data classification step, MBOSS uses the vector space model to measure the level of similarity between a non-labeled histogram and a labeled histogram. The main contributions of this study for HAR are: (1) the creation of a low cost, automatic process for feature extraction, i.e., one that does not depend on human knowledge; (2) a symbolic data model that combines the SFA noise tolerance and dimensionality reduction; (3) a classification model based on a vector space technique commonly used in the IR area; (4) a learning algorithm that provides the best compromise between classification accuracy and the use of computational resources, like memory and time processing.

MBOSS was evaluated through experiments conducted over three public databases, such as WISDM [17], UCI-HAR [18], and UniMib SHAR [19]. The evaluation strategies used were based on the personalized model (data from one user) and the generalized model (data from several users). MBOSS was compared with the HAR systems based on the manual feature extraction from the time and frequency domains used by conventional machine learning algorithms. The conventional algorithms evaluated were the K-Nearest-Neighbors, Decision Tree, Naïve Bayes, and Vector Support Machines. The results show good performance of the classifiers MBOSS in relation to other classifiers in terms of accuracy, processing time, and memory occupancy space.

The remainder of this article is organized as follows: Section 2 describes the symbolic representation algorithms used in this work. Section 3 describes the implementation of the proposed method. Section 4 describes the set of experiments and the results regarding the comparative analysis of the activity classifiers. Section 5 summarizes the main related work. Finally, Section 6 presents the conclusions and directions for future work.

## 2. Symbolic Representation Algorithms

This section describes the symbolic representation algorithms used for time series as the basis for developing the method proposed in this work. Previous studies [20] have shown the potential to extract discrete features from inertial sensors signals represented by histograms based on word frequency distribution. The main idea of these algorithms is to reduce data dimensionality and noise of the time series in order to improve the classification stage of the machine learning algorithms.

### 2.1. SFA: Symbolic Fourier Approximation

**Figure 1:**
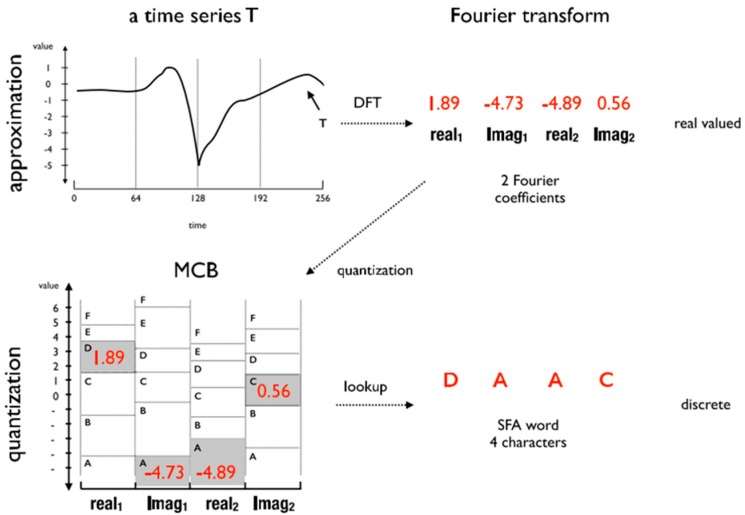
SFA Transform: A time series T is estimated using Fourier Transform and quantized using MCB lookup table. The result is a SFA word “DAAC” of length 4 and alphabet of size 6 [14].

The SFA [14] is a symbolic representation algorithm composed of two phases: approximation and quantization. The approximation phase consists of an application of the Discrete Fourier Transform (DFT) algorithm over the time series. The DFT results are real and imaginary coefficients that represent the time series in the frequency domain. This phase is based on the first l/2 coefficients of an instance, where l is the length of the word SFA. The quantization phase consists of selecting the most representative coefficients to generate the Multiple Coefficient Binning (MCB). The MCB is responsible for generating the words in the discretization process. Figure 1 shows an example of the SFA application for a time series of size n=256, word length l=4, and alphabet size c=6.

#### 2.1.1. Approximation Using the Discrete Fourier Transform (DFT)

The DFT [21] is an algorithm that decomposes a time series T of size n into a sum of basic orthogonal functions, for example, sine waves. Each wave is represented by a complex number $X_{u}=\left(real_{u},imag_{u}\right),$ for u=0, …, n−1, called Fourier coefficient. The *n*-value of Fourier coefficients of a time series T(x)=0,…, n−1 is given as the equation:

(1) $$DFT\left(T\right)=X_{0}\ldots X_{n-1}=\left(real_{0},imag_{0},\;\ldots \;real_{n-1},imag_{n-1}\right),$$

with

(2) $$X_{u}=\frac{1}{n}\sum_{x=0}^{n-1}T\left(x\right)\cdot e^{-j2\pi ux/n},\;\mathrm{for}\;u\in \left[0,n\right),\;j=\sqrt{-1}.$$

The first Fourier coefficients are related to the lower frequency ranges that represent slow changes in the signal. The higher order coefficients correlate to higher frequency bands representing rapid changes in the signal. The SFA uses the first Fourier coefficients because they are sufficient to roughly describe most of the signal. The DFT approximation is equivalent to the application of a low-pass filter for smoothing and removal of signal noise. The first coefficient $X_{0}=\frac{1}{n}\sum_{x=0}^{n-1}T\left(x\right)\cdot e^{0}$ is equal to the mean value of the signal, and can be discarded to obtain offset invariance (vertical shifts).

#### 2.1.2. Quantization Using the Multiple Coefficient Binning (MCB)

The quantization process consists of generating a search table in the time series, whose intervals define the letters that compose the word generated after the discretization process. This lookup table is called MCB (Multiple Coefficient Binning). The purpose of MCB is to minimize the loss of information introduced by the quantization process. The MCB intervals are calculated from an ordered array of Fourier coefficients obtained from the samples. The data in a matrix column are used to define the breakpoints that represent the interval between letters. The matrix rows are the first real and imaginary coefficients of the time series. The MCB is generated based on the following definitions:

*Quantization Intervals* (bins): the a-th quantization interval QI is defined by its upper β(a) and lower breakpoint β(a−1) and labeled by the *a*-th $symbol_{a}$ of the alphabet ∑:

(3) $$QI\left(a\right)=\left[\beta \left(a-1\right),\beta \left(a\right)\;\right)≜symbol_{a}\in \sum .$$

The number of bins corresponds to the number of values used in the DFT. An index *j* is used to denote the corresponding values of the Fourier Transform $DFT\;\left(T\right)=t_{0}^{\prime },\ldots \;,\;t_{j}^{\prime },\ldots ,\;t_{l-1}^{\prime }$:

(4) $$QI_{j}\left(a\right)=\left[\beta_{j}\left(a-1\right),\beta_{j}\left(a\right)\;\right)≜symbol_{a},\;j\in \left[0\ldots l\right),\;a\in \left[1\ldots c\right),\;\left|\sum \right|=c.$$

**Figure 2:**
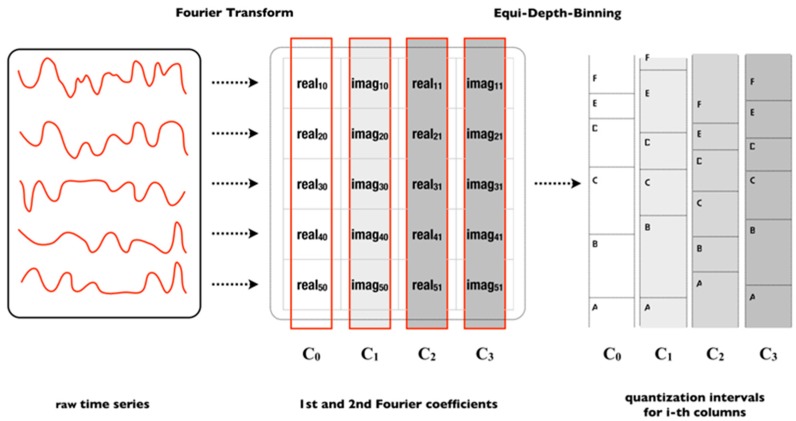
A set of time series is used to generate the bins and breakpoints in MCB. First, Fourier transform is applied and later is applied equi-depth binning for each *i*-th columns.

*Matrix MCB*: The matrix $A={\left(a_{ij}\right)}_{i=1,\;\ldots ,\;N;j=1,\ldots ,l}$ is constructed from the data of *N* approximate training time series ($T_{i,\;i\in \left[1,\ldots ,N\right]}$) with only the $\frac{l}{2}$ Fourier coefficients—equal to an SFA word of length l with $\frac{1}{2}l$ real and imaginary values. The i-th row of matrix A corresponds to the Fourier transform of the i-th samples $T_{i}$ (see Figure 2):

(5) $$A=\left(\begin{array}{c} DFT\left(T_{1}\right)\\ \vdots \\ DFT\left(T_{i}\right)\\ \vdots \\ DFT\left(T_{N}\right) \end{array}\right)=\left(\begin{array}{ccccc} real_{1;0} & imag_{1;0} & \cdots & real_{1;\frac{l}{2}-1} & imag_{1;\frac{l}{2}-1}\\ \vdots & \vdots & \cdots & \vdots & \vdots \\ real_{i;0} & imag_{i;0} & \cdots & real_{i;\frac{l}{2}-1} & imag_{i;\frac{l}{2}-1}\\ \vdots & \vdots & \cdots & \vdots & \vdots \\ real_{N;0} & imag_{N;0} & \cdots & real_{N;\frac{l}{2}-1} & imag_{N;\frac{l}{2}-1} \end{array}\right)=\left(C_{0}\;C_{1}\ldots \;C_{l-2}\;C_{l-1}\right).$$

The j-column $C_{j}$ corresponds to either the real or imaginary values of all N train samples. Each column is sorted by value and then partitioned into c equi-depth bins. Bins follow the equi-depth binning, in which the total amount of values in a range $\left[\beta_{j}\left(a-1\right)\leq \beta_{j}\left(a\right)\right)$ is equal to any other range.

The MCB quantization returns l sets of c quantization intervals for an SFA word. These intervals are given by:

(6) $$\mathrm{MCB}=\left(\begin{array}{ccccc} -\infty & -\infty & \cdots & -\infty & -\infty \\ \beta_{0}\left(1\right) & \beta_{1}\left(1\right) & \cdots & \beta_{l-2}\left(1\right) & \beta_{l-1}\left(1\right)\\ \cdots & \cdots & \cdots & \cdots & \cdots \\ \beta_{0}\left(c-1\right) & \beta_{1}\left(c-1\right) & \cdots & \beta_{l-2}\left(c-1\right) & \beta_{l-1}\left(c-1\right)\\ +\infty & +\infty & \cdots & +\infty & +\infty \end{array}\right).$$

The lookup table maps the first $\frac{l}{2}$ Fourier coefficients ($\frac{l}{2}$ real values and $\frac{l}{2}$ imaginary values).

*SFA Word*: The symbolic representation $SFA\;\left(T\right)=s_{0},\;\ldots ,\;s_{l-1}$ of the time series T with approximation $DFT\left(T\right)=real_{0},\;imag_{0},\ldots ,\;real_{\frac{l}{2}-1},\;imag_{\frac{l}{2}-1}=t_{0}^{\prime },\ldots ,\;t_{l-1}^{\prime }$ is mapping $SFA:\;{\mathbb{R}}^{l}\to \sum^{l}$ of a real value to a symbol over the alphabet $\sum =\left\{symbol_{1},\ldots ,\;symbol_{c}\right\}$ of size c. Specifically, the-*j* numeric value $t_{j}^{\prime }\in DFT\left(T\right)$ is mapped to a-th symbol of the alphabet ∑, if it corresponds to the interval $QI_{j}\left(c\right)=\left[\beta_{j}\left(a-1\right),\beta_{j}\left(a\right)\right)$:

(7) $$s_{j}=\{symbol_{a},\;\mathrm{if}\;\left(\beta_{j}\left(a-1\right)\leq t_{j}^{\prime }<\beta_{j}\left(a\right)\right),\;\mathrm{for}\;j\in \left[0\ldots l\right).$$

In the lower right-hand corner of Figure 1 the mapping and MCB quantization intervals are shown. As a result, an SFA word equal to “DAAC” is obtained from DFT(T)=(1.89, −4.73, −4.89, 0.56).

The SFA complexity is based on the discrete Fourier transform (DFT), where it is the dominant stage that consumes more processing. In order to optimize this step, the SFA was implemented using a DFT variation called Momentary Fourier Transform (MFT). The MFT is an evolution of the Fast Fourier Transform (FFT) [22]; it has a recursive property applied in the time windows. Further details on this optimization can be found in [16]. Therefore, the SFA has the following complexity:

(8) T(SFA)=O(nlogn),

with *n* as the time series size.

### 2.2. BOSS: Bag-Of-SFA-Symbols

The BOSS is a classification algorithm of time series based on symbolic data generated using SFA [16]. The BOSS data representation is based on word frequency distribution in the form of histograms. This data representation is obtained following a process of three tasks: windowing, symbolic transformation and histogram construction.

**Figure 3:**
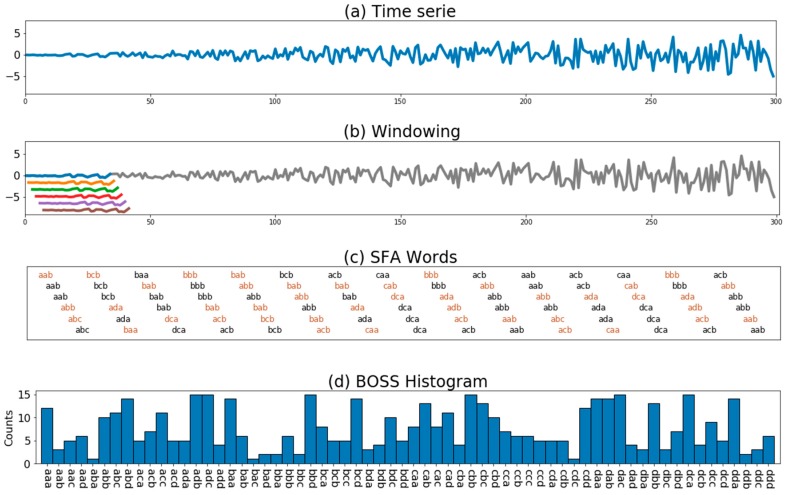
Example of the BOSS model construction: (**a**) a normalized time series; (**b**) windowing process to extract subseries; (**c**) symbolic transformation of SFA with numerosity reduction; and (**d**) histogram construction based on the repetition frequency of the SFA words.

Figure 3 illustrates the process of creating the BOSS model from a previously normalized size series n (mean zero and standard deviation equal to one). Firstly, the windows are obtained by applying a windowing procedure of size w along the time series (Figure 3b). The total number of windows obtained is (n−w+1). Then, each data window is submitted to symbolic transformation using the SFA algorithm (Figure 3c). Finally, the histogram of the SFA words (Figure 3d) is constructed. At this point, the numerosity reduction procedure is applied to reduce the repeated words in neighboring windows that often occur in stable signals. Thus, the SFA word counting only occurs in its first iteration, and the next repeated words are not counted in the histogram until a word change occurs (Figure 3c).

The BOSS complexity is based on the SFA complexity. The difference is in the time windows and the time series sizes. Thus, Equation (9) represents the BOSS complexity algorithm:

(9) T(BOSS)=O(n+wlogw)

where n is the time series size and w is the time window size.

### 2.3. BOSS VS: Bag-Of-SFA-Symbols in Vector Space

The BOSS VS algorithm is an extension of the BOSS algorithm combined with a classification strategy based on the vector space model [16]. The vector space template is commonly used by the information retrieval community to retrieve relevant documents based on a query formed by a set of terms or keywords. In short, the vector model means that the words of each document are represented in the n-dimensional space by a vector, in the same way the terms of the query are also represented by a vector. Thus, the document vectors are compared to the query vector by measuring similarity. Therefore, the query vector is sorted by the document whose vector is closest or similar.

Each vector has an associated weight. Weights are assigned to each document term and query. Both term frequency (*tf*) and inverse document frequency (*idf*) techniques [23] help to distribute weights so that larger weights are assigned to the most representative terms, whereas irrelevant terms receive smaller weights.

The BOSS VS model is constructed according to the approach described by Senin & Malinchik [24], in which the *idf* is calculated for the words of each class as opposed to each time series. The *tf* for an SFA word p of a time series T is given by the following equation:

(10) $$tf\left(p,T\right)=\{\begin{array}{c} 1+\log\left(B_{T}\left(p\right)\right),\;\mathrm{if}\;B_{T}\left(p\right)>0\\ 0,\;\mathrm{else} \end{array},$$

where $B_{T}\left(p\right)$ being the BOSS histogram, which represents the frequency of an SFA word *p* in the time series *T*. Therefore, the *tf* for an SFA word p in a class C is given by the following equation:

(11) $$tf\left(p,C\right)=\{\begin{array}{c} 1+\log\left(\sum_{T\in C}B_{T}\left(p\right)\right),\;\mathrm{if}\;\sum_{T\in C}B_{T}\left(p\right)>0\\ 0,\;\mathrm{else} \end{array}.$$

The *idf* of a word p in the C class is calculated by the following equation:

(12) $$idf\left(p,C\right)=\log\frac{\left|CLASSES\right|}{\underset{number\;of\;classes\;that\;contain\;p}{\underset{︸}{\left|\{C|\;T\in C⋀B_{T}\left(p\right)>0\text{\}}\right|}}},$$

where idf(p, C) is the total number of classes divided by the number of classes in which the word p occurs. A high *idf* value is obtained when the word occurs in only one specific class, meaning that it is a relevant term for the discrimination of a given class. On the other hand, a word with low *idf* is common to several classes, meaning that word is not relevant.

Finally, the *tf-idf* of an SFA word in a C class is calculated by the following equation:

(13) tf-idf(p,C)=tf(p,C)·idf(p,C).

Classification consists of inferring an activity based on the comparison between a set of words from a query Q and a set of words from a class (or activity) C. Both sets of words are represented by a vector generated from *tf-idf*. The comparison between two vectors is performed by calculating the cosine similarity using the weights matrix *tf-idf* for each class C. Finally, the label of query Q is inferred by assigning the label of class C that maximized the result of the cosine similarity. Since the calculation of the cosine similarity is defined by the following equation:

(14) $$similarity\left(Q,\;C\right)=\frac{\vec{Q}\;.\;\vec{C}}{\left\|\vec{Q}\|.\|\vec{C}\right\|}=\frac{\sum_{p\in Q}tf\left(p,\;Q\right)\;.\;tf-idf\left(p,\;C\right)}{\sqrt{\sum_{p\in Q}{\left(tf\left(p,\;Q\right)\right)}^{2}}\sqrt{\sum_{p\in C}{\left(tf-idf\left(p,\;C\right)\right)}^{2}}}$$

And the label inferred by the following equation:

(15) $$label\left(Q\right)=argmax_{C\in CLASSES}\;\left(similarity(Q,C\right)).$$

In practice, the classification task is performed using the nearest 1-neighbor algorithm (1-NN). The computational complexity of the classification given by a 1-NN search over |CLASSES| classes is:

(16) T(BOSS VS)=|CLASSES|·O(n),

where n is the time series size. In general, the main advantage of the BOSS VS algorithm is scalability, since this algorithm is able to manipulate a large amount of data due to its ability to reduce a set of histograms that represent the same class in a more compact histogram only. This process significantly reduces the computational complexity of the model, resulting in a reduction of the classification times for new samples.

## 3. MBOSS: Multivariate Bag-Of-SFA-Symbols

MBOSS was designed to classify multiple time series (or multivariate time series) because of the three-dimensional characteristic of the inertial sensors embedded in smartphones. The multivariate time series are collections of univariate time series that arise when multiple interconnected data sources capture samples over time [25]. The MBOSS is an extension of the BOSS algorithm and is mainly characterized by the histograms fusion generated by multiple synchronized time series.

### 3.1. Stacking Histograms

**Figure 4:**
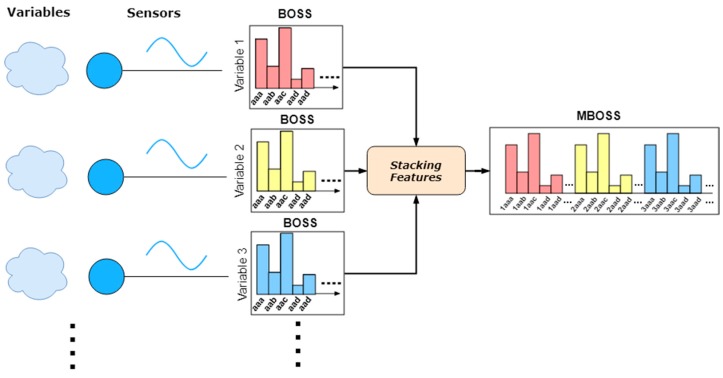
Data fusion at the features level: Example of concatenation of histograms (Feature Stacking).

MBOSS uses a concatenation function that combines multiple histograms generated by the BOSS. Like several other research areas, as image recognition, the stacking of features strategy is used [26]. Figure 4 shows an example of the histogram fusion process. Basically, the fusion is based on concatenation of M histograms of dimension N (number of variables) into a single feature vector. After that, the final feature vector is used for the training and classification task.

The pseudocode of Algorithm 1 shows the MBOSS implementation. This algorithm receives a single structure of multivariate time series as input. This structure is defined as a time-series vector $s=\left\{s_{1},\ldots ,\;s_{i},\ldots ,\;s_{v}\right\}$ where $s_{i}$ corresponds to an input time series in the variable i, and v the vector size (line 1). The algorithm output is defined by a structure representing the histogram (line 2). The algorithm transforms the time windows into symbolic data for each variable (line 3) and constructs the histogram from the SFA words. MBOSS maintains the information of the original time series that generated the words using an index (line 5). For example, if a word “ab” extracted from variable 2 will receive the character “2”, then the new word formed will be “2ab”. Finally, the numerosity reduction is applied (lines 6–8), and the algorithm result is the MBOSS histogram (line 8).

**Algorithm 1.** MBOSS Transformation.1**function MBOSSTransform** (MultivariateTimeSerie s, **int** v, **int** w, **int** l, **int** c)2 map<String, **int** > histogram = [], String lastWord = NULL3 **for int** i_v in [1..v]4  **for** TimeSeries window in sliding_windows(s[i_v], w) // sliding window5   String word = **toChar**(i_v) + SFA(window, l, c) // SFA word + variable identifier6   **if** word != lastWord         //numerosity reduction7    histogram[word]++8   lastword = word9 **return** histogram

The MBOSS complexity is based on the BOSS complexity. The difference is the number of input variables, as shown in the following formulas:

(17) T(MBOSS)=v·T(BOSS)

T(MBOSS)=v·O(n)

T(MBOSS)=O(n), with v≪n,

with n as the size of time series and v≪n means that the number of variables is much smaller than the segment size.

### 3.2. Classifier for HAR

**Figure 5:**
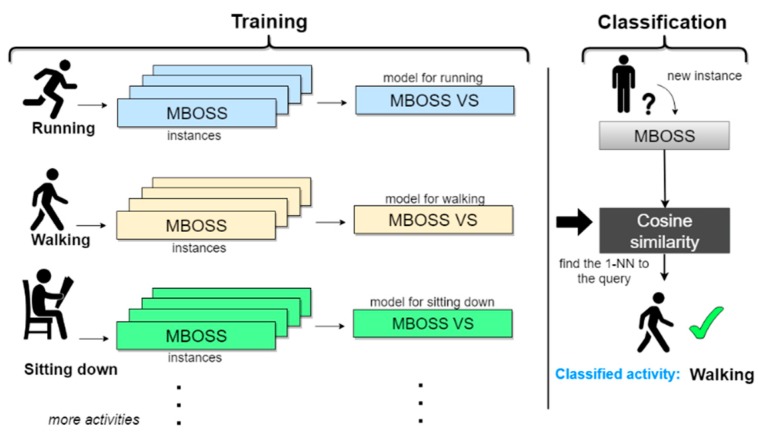
MBOSS VS classifier based on the vector space model to classify human activities.

Figure 5 illustrates the activities classification scheme using the MBOSS data representation in the vector space model. In this example, the data from three activities (running, walking, and sitting down) are used to generate the MBOSS histograms. Then, the histograms are represented by matrices generated through the vector model. This new data representation is called MBOSS VS. The MBOSS VS model is generated based on the *tf-idfs* calculations explained in Section 2.3. Each MBOSS VS model is a compact representation of all training instances of a given activity class. The vectors *tf-idf* correspond to each class and are used to infer the label of an unknown instance through the calculation of the cosine similarity between the vectors *tf-Idfs* and the vector *tf* obtained from the unknown instance (Equation (10)). Based on these calculations, the unknown instance is assigned to a class label that maximizes the cosine similarity result.

Algorithm 2 shows the pseudocode of MBOSS VS. Line 1 consists of the input parameters referring to the matrix structure of *tf-idfs* and *tf* vector from unknown instance. Then, the cosine similarity is calculated based on the dot-product between the *tf-idfs* and *tf* vectors (rows 4–5). Finally, the output label refers to the class that maximized the result of the cosine similarity (lines 6–9).

**Algorithm 2.** Classification using 1-NN and MBOSS-VS models.1**function predict**(map<String, **int**> tf, map<String, **int**>[] tfIdfs)2 **double** maxSim = 03 String bestClass = NULL4  **for int** classId in [1..len(tfIdfs)] // do for each class5   **double** cosSim = dotProduct( tf, tfIdfs[classId]) // similarity calculation6   **if** cosSim > maxSim7    maxSim = cosSim8    bestClass = classId9 **return label**(bestClass)

The vector product used to calculate the cosine similarity has computational complexity equal to O(n), where n is the vector size of the instance that was submitted to classification. Therefore, the classification 1-NN MBOSS VS (Algorithm 2) has computational complexity equal to:

(18) T(1-NN MBOSS VS)=|CLASSES|·T(similarity)

T(1-NN MBOSS VS)=|CLASSES| ⋅O(n),

with n as the vector size of the unknown instance and |CLASSES| as the number of classes.

### 3.3. Optimizing MBOSS Parameters

In the feature extraction step, three parameters must be adjusted to use MBOSS: window size w, word length l, and alphabet size c. These parameters directly influence the performance of the generated classifier and must be adjusted according to the problem. Thus, the values of these parameters are defined below:

- Window size: it is defined based on the analysis of the length w in the intervals ranging from 10 to n (n is the time series size). To reduce training times, only the $\sqrt{n}$ windows are used.
- Word length: it is related to the first l/2 DTF coefficients used to approximate the signals. A small word length is desirable, which correlates to a strong noise reduction (low-pass filter). If it is use larger values, the ability of SFA’s noise reduction is degraded. In the context of HAR smartphone-based, a set of values ranging from 4 to 16 is used.
- Alphabet size: it is related to the number of intervals in the SFA’s quantization phase. The larger the alphabet size, the greater the ability of the method to approximate to the original time series. However, Schäfer [16] claims that the alphabet size c=4 is enough to approximate any time series, since a large alphabet size and large word length consume more computing resources. In a 32-bit processing platform, the parameters c=4 and l=16 generate $4^{16}=32^{2}=4,294,967,296$ possible words. Any other option must meet the requirements of the processing platform to be implemented, observing the maximum possible number of words that can be represented.

The parameter optimization process is implemented by Algorithm 3 and is based on the grid search with cross validation, with k=10 partitions, to estimate the best values of w and l. The algorithm iterates all $\sqrt{n}$ window sizes (line 5). For each iteration, the MBOSS model is obtained for different window sizes (lines 7 and 8). Then, the algorithm iterates over the word length options, in this case l∈{4, 6, 8, 10, 12, 14, 16} (line 9). For each word size, the symbolic representations of each segment sample with word size l are constructed. In order to avoid recalculating the MBOSS transformation for different word sizes, the larger word l=16 is firstly calculated, and it is used as the basis for reconstructing other, smaller words (line 10). 

The cross validation is applied to obtain the combination w and l that obtained the highest rate of accuracy in the training step. This is performed by dividing the samples into ten partitions. Next, one partition is selected and used as the test dataset. The remaining nine partitions are used to construct the classification model. This process is repeated until all partitions are evaluated and the classification results for each partition are combined to obtain a final score. Finally, cross-validation is repeated until all parameter combinations are evaluated, so that the output of the algorithm are the best estimated values of l and w.

**Algorithm 3.** Fit MBOSS using *10-fold cross-validation*.1**function fit**(MultivariateTimeSerie[] samples, **int** n, **int** v, **int** folds)2 **int** c = 4, **int** maxL = 163 **int** bestScore = 0, **int** bestL = 0, **int** bestW = 034 map<String, int > bestTfIdfs = []5  **for** (**int** w = 10; w <= in; w += sqrt(n-10))   *// itera sqrt(n)*6   map<String, **int** > sHist = []7   **for int** i in [1..len(samples)] *// N*8    sHist[i] = MBOSSTransform(samples[i], v, maxL, c) *// O(n)*9   **for int** l in [maxL.., 8, 6, 4]10    map<String, **int** >[] bags = createBags(sHist, l)11    **int** score = 012    **for int** i_fold in [1..folds] *// cross validation*13     map<String, **int** >[] bags_test, bags_train, tfIdfs14     bags_test = getFold(i_fold, bags)15     bags_train = getOthersFold(i_fold, bags)16     tfIdfs = calcTfIdf(bags_train, l)17     score += predict_score(bags_test, tfIdfs_train)18    **if** score > bestScore19     bestScore = score, bestL = l20     bestW = w, bestTfIdfs = tfIdfs21 **return** (bestScore, bestL, bestW, bestTfIdfs)

The MBOSS parameters optimization (Algorithm 3) has computational complexity equal to:

(19) $$T\left(FIT\;MBOSS\right)=\sqrt{n}\cdot N\cdot \left[T\left(MBOSS\right)+T\left(1-NN\;MBOSS\;VS\right)\right]$$

$$T\left(FIT\;MBOSS\right)=\sqrt{n}\cdot N\cdot \left[v\cdot O\left(n\right)+\left|CLASSES\right|\cdot O\left(n\right)\right]$$

$$T\left(FIT\;MBOSS\right)=O\left(N\cdot v\cdot n^{\frac{3}{2}}\right),$$

where n is the size of the multivariate time series, v is the number of variables and N is the amount of time series.

## 4. Experiments and Results

This section presents the experiments performed to evaluate MBOSS in the context of the HAR problem. In this study, MBOSS is evaluated from two perspectives. The first one deals with a comparative analysis between classification models designed for HAR. The second one deals with an analysis of the consumption of computational resources required by the evaluated methods. It includes the data processing time and memory usage analysis. The databases used in the experiments are described in Section 4.2, and the results for each analysis are described in Section 4.5.

### 4.1. Experimental Protocol

The experiments are divided into two steps:

1. Comparative analysis of the classification models: the comparative analysis includes the comparison between MBOSS classifier and the baselines classifiers described in Section 4.3. Two strategies of classification model evaluation are used: personalized model and generalized model. These strategies exploit different experimental configurations that are often found in the literature, and provide a comprehensive analysis of the classifiers.
2. Comparative analysis of the time processing and memory consumption: The time processing analysis occurs in three stages: feature extraction, training, and testing of the classification models. These results show the viability of using MBOSS in mobile devices. The memory consumption analysis evaluates the capacity of the MBOSS to reduce the amount of data in terms of data compacting.

### 4.2. Datasets

Table 1 summarizes the characteristics of the datasets used in the experiments. The main characteristics highlighted are the number of users, number of activities (classes), and segmentation settings that were used in the data pre-processing. The WISDM, UCI HAR, and UniMiB SHAR datasets have 176, 343 and 253 instances per user, respectively, and the segmentation configurations are identical to those described by the authors in the respective original published articles.

The WISDM dataset was provided by the Wireless Sensor Data Mining laboratory of the Department of Computer Science at Fordham University [17]. This dataset contains accelerometer data from different Android smartphones like Nexus One, HTC Hero, and Motorola Back-flip. During data collection, the user was asked to carry the device in one of their front pants pockets. Data was collected from 36 users for six physical activities: walking, jogging, walking downstairs, walking upstairs, sitting, and standing. However, in our experiments we only selected the users that had instances of all six activity-classes. Samples from each user were collected at a fixed sampling rate of 20 Hz. Data segments were obtained every 10 s by applying the sliding window algorithm with fixed size equal to 200 samples per segment without overlap between windows. In total, the dataset contained 3345 segments.

The UCI-HAR dataset was provided by the Machine Learning Repository of the University of California at Irvine (UCI) [18]. This database contains data from the inertial sensors accelerometer and gyroscope collected from an Android smartphone Galaxy SII. The data was collected from 30 users for six physical activities: walking, walking upstairs, walking downstairs, sitting, standing, and lying down. Samples from each user were collected at a fixed sampling rate of 50 Hz. Users executed the activities in two sessions. In the first session, users were asked to carry the smartphone on the left side of the waist, and in the second session, users were asked to carry the smartphone in a free position. In our experiments, we used data from all users and only those from the accelerometer. Data segments were obtained every 2.56 s, applying the sliding window algorithm with fixed size equal to 128 samples per segment with 50% of overlap between windows. In total, the dataset contained 10,299 segments.

The UniMib SHAR dataset was provided by the IT department at the University of Milano-Bicocca [19]. This dataset contains accelerometer data obtained from a smartphone, the Android Galaxy Nexus I9250. The data was collected from 30 users for nine physical activities and eight activities related to fall events. Users were selected based on attributes such as height (169 ± 7 cm), weight (64 ± 10 kg), age (18 to 60 years), and gender (24 women and 6 men). Samples from each user were collected at a fixed sampling rate of 50 Hz. Users executed the activities in two sessions. In the first session, users were asked to carry the smartphone in the front left pocket of the pants, and in the second session, the smartphone was carried in the right pocket. Only data related to physical activities was used in our experiments.

The UniMib SHAR has particular data segmentation characteristics based on the magnitude $mag\;\left(t\right)=\sqrt{a_{x}{\left(t\right)}^{2}+a_{y}{\left(t\right)}^{2}+a_{z}{\left(t\right)}^{2}}$ of the accelerometer signal. This method consists of applying the sliding window technique to extract 3 s segments every time a peak in magnitude is encountered. A peak is valid only if it reaches certain conditions: (1) The signal magnitude $mag_{t}$ is greater than 1.5g, being g, the value of the universal gravitation constant; (2) The signal magnitude $m_{t-1}$ at the instant t−1 is less than 1.5g. These conditions are justified by the authors to classify both movement and fall activities. Each segment of 3 s is centered on the peak found. In total, the dataset contained 7579 instances.

### 4.3. Baseline

The MBOSS baselines are conventional machine learning algorithms as Decision Tree (J48), Naive Bayes (NB), K-Neighbors (KNN) with k=1, and Support Vector Machines (SVM) implemented with SMO One-vs-One with polynomial kernel. The experiments were executed in the WEKA tool (Waikato Environment for Knowledge Analysis) [28]. Each algorithm was trained with a set of features extracted from the Time and Frequency domain (Hand-Crafted Features). The selected features were obtained based on previous work found in the literature [29,30]. Table 2 presents the features selected for the experiments. In total, 36 features were obtained from the tri-axial accelerometer data, i.e., 12 features for each axis x, y, z.

### 4.4. Evaluation Strategies

Different strategies are used in the literature to evaluate HAR systems [31]. This experiment uses two common strategies. The first strategy is the generation of a personalized model, and the second is the generation of a generalized model:

1. **Personalized model**: consists of creating a model for a single user. The evaluation process is based on a cross-validation technique where the data user is partitioned into 10-fold, so that each part is used as the test set, and the remaining nine parts are used as a training set. Previous studies identify this strategy as a personal model, or user-dependent model.
2. **Generalized model**: consists of creating a generic model based on data from several users. The evaluation process is based on a cross-validation technique where each partition is represented by the data from different users. Basically, a target user n is used as the test set, and the remaining users (n−1) are used as the training set. In the literature, this type of assessment is also called “cross-validation leave-one-subject-out” or “user-independent model”.

The consolidated results of the evaluations are presented using accuracy as the main metric, and the confusion matrix for analysis of each activity class.

### 4.5. Classification of Effectiveness Analysis

This section presents the results in two parts: the first part is related to the personalized model and the second part to the generalized model.

#### 4.5.1. Personalized Model

**Figure 6:**
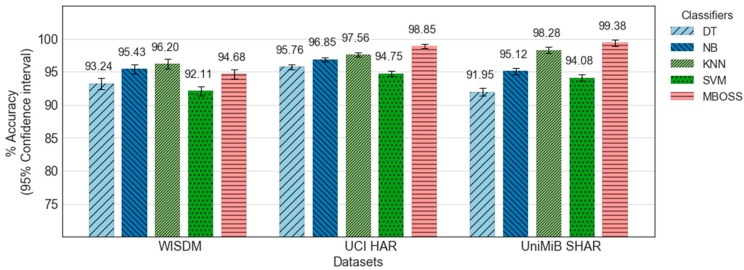
Accuracy results for all classifiers evaluated based on personalized model.

**Figure 7:**
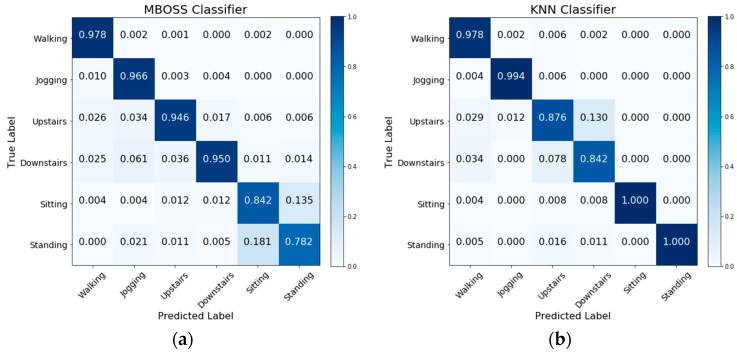
Confusion matrices for (**a**) MBOSS and (**b**) KNN classifiers for the WISDM dataset.

Figure 6 shows the comparative analysis results between MBOSS and the other baselines for the personalized model. As presented, the results show that MBOSS obtains the best accuracy rates for the UCI-HAR and UniMiB SHAR datasets, with 98.85% and 99.37%, respectively. For the WISDM dataset, the KNN classifier obtained the best performance, with an accuracy of 96.2%, i.e., 1.53% higher than the accuracy of MBOSS. To clarify this difference, the confusion matrices for the MBOSS and KNN are presented in Figure 7.

The results for each activity class show that MBOSS have lower hit rates than the KNN for static activities such as sitting and standing in the WISDM dataset. However, this difference is not significant in the other databases. One reason for this difference is the segmentation settings. In UCI HAR and UniMiB SHAR datasets, each instance is composed of data collected at 50 Hz over a short period, around 3 s. On the other hand, in WISDM dataset each instance is composed of data collected at 20 Hz over a long period, 10 s. Therefore, high sampling over short periods is more favorable for symbolic representation.

#### 4.5.2. Generalized Model

**Figure 8:**
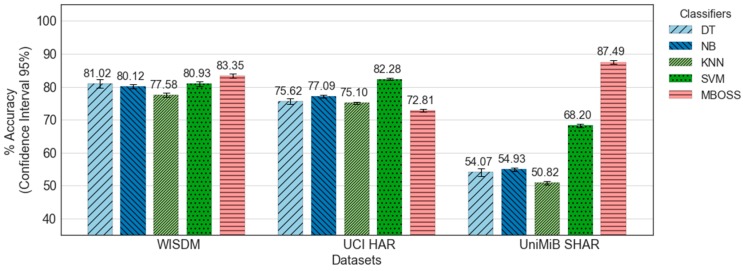
Accuracy results for all classifiers evaluated based on generalized model.

Figure 8 shows the comparative analysis between MBOSS and the other baseline classifiers for the generalized model. As presented, the results show that MBOSS obtains the best accuracy rates for the WISDM and UniMiB SHAR datasets, with 83.35% and 87.49%, respectively. For the UCI HAR database, the SVM classifier obtained the best performance, with an accuracy of 82.28%, i.e., 19.29% higher than the accuracy of the MBOSS. On the other hand, the results for the UniMiB SHAR show that the baseline classifiers obtained unsatisfactory accuracy rates. One reason for this is that UniMiB SHAR is an unbalanced dataset with a large number of activity classes. In this case, MBOSS obtained an accuracy of 87.49%, and remained stable for different experimental settings.

**Figure 9:**
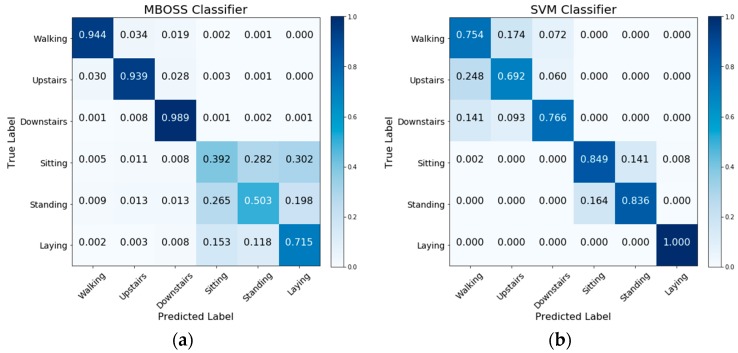
Confusion matrices for (**a**) MBOSS and (**b**) SVM classifiers for the UCI HAR dataset.

Figure 9 presents the confusion matrices obtained from the MBOSS and SVM classifiers for UCI HAR dataset. The analysis by class shows that the accuracy of the MBOSS, in general, decreases for a specific set of static activities like sitting, standing, and laying down. On the other hand, MBOSS presents the highest accuracies for dynamic activity classes like walking, and walking up- and down- stairs.

#### 4.5.3. Comparison between Strategies

**Figure 10:**
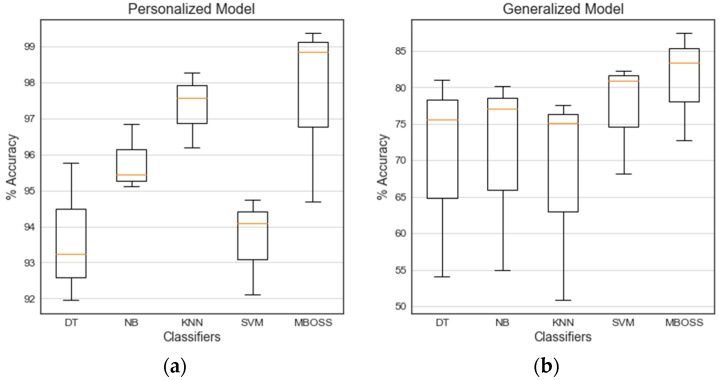
Classification results of (**a**) the personalized model and (**b**) the generalized model.

Figure 10 shows the boxplot diagrams consolidating the classification results for each evaluation strategy. Note that the results for all classifiers in the personalized model have satisfactory accuracy rates, with values between 92% and 100%. In this evaluation, MBOSS demonstrated performance comparable to baselines with a median accuracy of 98.85%, i.e., 1.29% higher than the KNN.

In addition, the accuracies of the generalized model were below 90%. This low performance is expected when compared to personalized model, because it is difficult to generate generic and flexible models for different users’ profiles. However, in this context the MBOSS presented a higher generalization capacity compared to the baseline classifiers. On average, the MBOSS has a median accuracy of 83.34%, i.e., 1.06% higher than the SVM classifier.

### 4.6. Results of Time Processing and Memory Consumption Analysis

This section presents two types of results. The first type deals with a comparative analysis between the processing time of the MBOSS and the baselines algorithms. The second deals with an analysis of the memory consumption of the MBOSS. The experiments were executed on a Dell OptiPlex 9020 Desktop Computer (Intel Core i7-4790/32GB DDR3). The database used in this experiment was UCI HAR, in which the data was partitioned into two sets: a training set with data from 21 users, and a testing set with data from 9 users.

#### 4.6.1. Time Processing

The data processing times of the classification algorithms were collected in three steps:

1. Feature extraction: processing time required to generate the set of features on the test instances.
2. Classification model training: processing time required to generate the classification model on the training set.
3. Classification: processing time required to classify only the test set.

**Figure 11:**
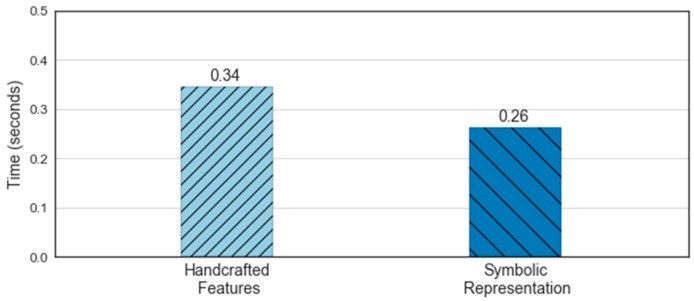
Comparison of computation time for extraction features.

Figure 11 shows the processing times for the feature extraction step from the time and frequency domain (baseline) and the MBOSS. The results are based on the average of ten-run times. As presented, the feature extraction based on symbolic representation data has slower processing time than the baseline, with a difference of 0.08 s. This represents a time reduction of 23.5%. Although we used a small set of features, the symbolic representation shows rapid processing of the sensor data; therefore, it is more efficient.

**Figure 12:**
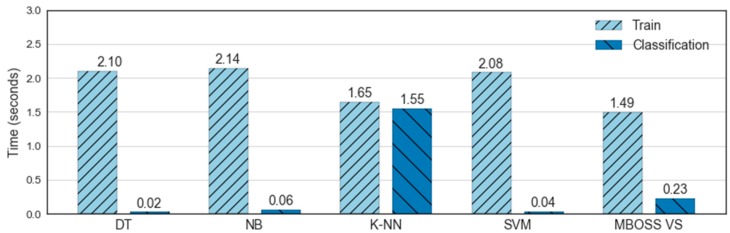
Comparative computational time for the training and classification of human activities.

Figure 12 shows the processing times for the classifier training and testing steps. As presented, the results show that MBOSS took 1.49 s for the training process and 0.23 s for the testing process. It shows that the MBOSS obtained the shortest time to train the classification model, with a difference of 0.16 s compared to the KNN. On the other hand, in the testing step the MBOSS took longer than the classifiers DT, NB, and SVM, with a difference of about 0.20 s. This occurs because these algorithms present a computational cost in the order of O(d), where d is the number of features. On the other hand, MBOSS beat KNN because it had a computational cost for classification step in the order of O(Nd), where N is the number of training instances, and MBOSS has a computational cost in the order of O (cd), where c is the number of classes.

#### 4.6.2. Memory Consumption

The evaluation of the MBOSS memory consumption is based on the comparison between the data volume in the memory before and after the feature extraction process. The experiments were based on 10,000 instances obtained from the UCI HAR dataset. Each instance corresponds to a time series of 128 samples. Originally, each sample is represented in the memory by 8 bytes (double). Therefore, an original instance corresponds to a total of 1024 bytes. In addition, the results also showed the influence of reduced data numerosity on memory consumption. The size of memory consumed is evaluated in Kbytes.

**Figure 13:**
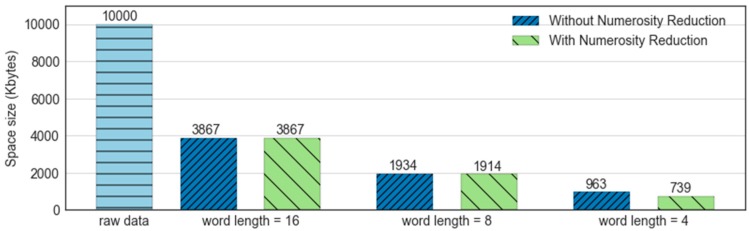
Comparison of the cost of memory space required for the processing of time series.

Figure 13 presents the results referring to the space consumed by the MBOSS symbolic data with the following parameters: alphabet size c=4, window size w=30, and word length l={4, 8, 16}. As presented, the result shows that the symbolic data can reduce the memory consumption from 60% to 90% over the space consumed by the original data. In addition, data reduction is directly influenced by word length, where a smaller word length corresponds to a larger data reduction after data processing.

## 5. Related Works

This section presents a summary of the main works that use the manual feature extraction strategy, and the works that use symbolic representation algorithms for the HAR solutions.

Kwapisz et al. [17] present a solution based on the manual feature extraction approach. In this work, the time domain features, as mean, standard deviation, absolute mean, time interval between signal peaks, and binned distribution, were used. The experiments were conducted using the accelerometer data extracted from the WISDM dataset. The classifiers used were the machine learning algorithms Decision Tree, Logistic Regressor, and a Multi-layer Perceptron Neural Network (MLP). Cross-validation with the partitioning of all instances of activities into 10 folds was the evaluation strategy used. Finally, the results showed the superiority of the MLP classifier in relation to the other classifiers evaluated, with an accuracy rate of 91.7%.

Anguita et al. [18] present a solution based on the manual feature extraction approach. In this case, 17 features were used in the time and frequency domain. These features were applied on the raw and preprocessed signals obtained from the accelerometer and gyroscope. The preprocessed signals were obtained from the application of digital filters, such as Butterworth low-pass, for the decomposition of acceleration between body signals and gravity. In addition, the signals were preprocessed using the magnitude and time derivatives of the linear and angular acceleration. The experiments were conducted in the UCI HAR database. The Support Vector Machine (SVM) One-vs-All was used as the classification algorithm, and evaluation consisted of partitioning the data 21-9, i.e., the data from 21 users was used for training, and the data from 9 users was used for testing. The results showed an average accuracy of 96% for the test data.

Figo et al. [29] present an exploratory evaluation involving the time, frequency, and discrete domains features. In the case of the discrete domain, data was discretized by the symbolic representation algorithm called SAX (Symbolic Aggregate Approximation). The features generated from the symbolic data were based on the distance between the words of each label. The experiments were conducted using accelerometer data, and jumping, running, and walking activities were studied. The classification stage is based on two steps. The first is to calculate the signal magnitude for each coordinate axis ($x_{i},\;x_{y},\;x_{z}$) using the formula $n_{i}=\sqrt{x_{i}^{2}+y_{i}^{2}+z_{i}^{2}}$. The second step is to find a threshold that separates the classes well, based on the evaluated features. For the time domain features (e.g., mean, minimum, maximum, and standard deviation), the results showed an average of about 70.4% for the training set and 60.8% for the testing set. For the features in the frequency domain, the results showed an average of 69.6% and 70.1%, with the best accuracy obtained by the feature sum of the spectral coefficients. For the features in the discrete domain, the results showed an average of 62.4% for the training set and 45.1% for the testing set.

Siirtola et al. [32] propose a solution based on the symbolic representation algorithm called SAXS (Symbolic Aggregate Approximation Similarity). SAXS is characterized by generating a set of reference words called templates, in which each one of them represents a label or activity. These words are defined during the classification model training step. The SAXS classification process is based on the calculation of similarity between the templates and the new, non-labeled words. Experiments were conducted on five accelerometer databases including gestural, sports, and swimming style activities. The classifiers used were Decision Tree, K-Nearest Neighbor (KNN), and Naive Bayes. The evaluation was based on the combination of the SAXS features with the time (mean, standard deviation, median, quartiles, minimum, and maximum) and frequency (sums of smaller sequences of Fourier-transformed signals, and number and ratio of zero crossings) domain features. The results showed that the features generated by the SAXS obtained better classification models, with an average accuracy rate between 3% and 10% when compared to the classification models generated only with the time domain and frequency features. The KNN classifier obtained the highest accuracy rate, i.e., 85.3%.

Finally, Terzi et al. [33] propose a method based only on the discrete domain features specialized in dealing with multivariate time series. The activities classification is performed by the 1-NN classifier with three similarity metrics: average, minimum, and geometric distance. In addition, the proposed method generates word templates and uses the SAX algorithm in the discretization process. The experiments were conducted using the accelerometer data obtained from the UCI HAR database for the following activities: walking and climbing stairs. The results showed that the similarity metric based on the average distance performed better than the other distances. The accuracy rate obtained was 99.54% and 97.07%, respectively, for the two studied activities.

The work of Siirtola and Terzi uses symbolic representation algorithms in the feature extraction step, as previously described. In general, in these works, the values corresponding to the similarity between pairs of words are calculated. Unlike these approaches, MBOSS extracts multiple words from the instances, counts the words, and generates models based on the word frequency distribution-histogram. This symbolic representation approach enriches the extracted knowledge and makes the classification models more precise.

## 6. Conclusions

This paper proposes a new time series classification algorithm (MBOSS) from the perspective of the HAR area. MBOSS is a low-cost algorithm that can be deployed on mobile devices such as smartphones. The main advantage of MBOSS is the reduction of computational complexity through the reduction of data dimensionality and numerosity. In addition, MBOSS is able to reduce data noise and extract features automatically. This reduces the influence of human knowledge in the configuration steps.

The experiments’ results show good performance of MBOSS for both evaluation strategies of the personalized and generalized models compared to the traditional approaches of manual feature extraction in the field of time and frequency. In particular, MBOSS’s impersonal models are able to thoroughly generalize for multiple activity categories. In addition, results regarding processing time and memory consumption show that MBOSS is generally the best low-cost option among the currently-available options. The results were extracted from different scenarios provided by the different databases with different segmentation settings.

The conclusions obtained from this work help motivate future work and develop new projects for HAR systems using inertial sensors in smartphones. Future work will be conducted to evaluate the performance of MBOSS in applications that use data collected from a smartwatch. In addition, new adaptations of symbolic representation algorithms, such as applying filters and feature selection techniques, can be evaluated.

## Figures and Tables

**Table 1:** Summary of dataset characteristics used in experiments [27].

Dataset	WISDM	UCI HAR	UniMib SHAR
Number of users	19	30	30
Number of activities	6	6	9
Segmentation technique	Sliding window without overlap	Sliding window with 50% of overlapping	Energy-based segmentation
Segment length	200 (10 s–20 Hz)	128 (2.56 s–50 Hz)	151 (3 s–50 Hz)
Total of instances	3345	10,299	7579

**Table 2:** Set of the features used in the experiments with the baseline classifiers.

Domain	Signal	Feature
Time	*x*-, *y*- *e z*-axis of accelerometer	mean, median, min, max, variance, standard deviation, zero crossing rate, and root mean square
Frequency	*x*-, *y*- *e z*-axis of accelerometer	dc component, sum of five first coef. of Fourier, spectral energy, and spectral entropy
